# Machine learning algorithm based on combined clinical indicators for the prediction of infertility and pregnancy loss

**DOI:** 10.3389/fendo.2025.1544724

**Published:** 2025-07-18

**Authors:** Rui Zhang, Yuanbing Guo, Xiaonan Zhai, Juan Wang, Xiaoyan Hao, Liu Yang, Lei Zhou, Jiawei Gao, Jiayun Liu

**Affiliations:** Department of Clinical Laboratory Medicine, Xijing Hospital, Fourth Military Medical University, Xi’an, China

**Keywords:** infertility, pregnancy loss, machine learning, 25OHVD3, diagnosis

## Abstract

**Background and objectives:**

Diagnosis and treatment of infertility and pregnancy loss are complicated by various factors. We aimed to develop a simpler, more efficient system for diagnosing infertility and pregnancy loss.

**Methods:**

This study included 333 female patients with infertility and 319 female patients with pregnancy loss, as well as 327 healthy individuals for modeling; 1264 female patients with infertility and 1030 female patients with pregnancy loss, as well as 1059 healthy individuals for validating the models. The average age and basic information were matched between the groups. Three methods were used for screening 100+ clinical indicators, and five machine learning algorithms were used to develop and evaluate diagnostic models based on the most relevant indicators.

**Results:**

Multivariate analysis revealed significant differences in several factors between the patients and the control group. 25-hydroxy vitamin D3 (25OHVD3) was the factor exhibiting the most prominent difference, and most patients presented deficiency in the levels of this vitamin. 25OHVD3 is associated with blood lipids, hormones, thyroid function, human papillomavirus infection, hepatitis B infection, sedimentation rate, renal function, coagulation function, and amino acids in patients with infertility. The model for infertility diagnosis included eleven factors and exhibited area under the curve (AUC), sensitivity, and specificity values higher than 0.958, 86.52%, and 91.23%, respectively. The model for potential pregnancy loss was also developed using five machine learning algorithms and was based on 7 indicators. According to the results obtained from the testing set, the sensitivity was higher than 92.02%, the specificity was higher than 95.18%, the accuracy was higher than 94.34%, and the AUC was higher than 0.972.

**Conclusion:**

The simplicity, good diagnostic performance, and high sensitivity of the models presented here may facilitate early detection, treatment, and prevention of infertility and pregnancy loss.

## 1 Introduction

Infertility is a condition defined as being unable to conceive after having regular unprotected sex for at least one year (1). Previous reports have indicated that 17% of women and 9.4% of men in the USA have used an infertility service (2). Infertility not only affects the mental health of the afflicted individual but is also a potential risk factor for many cancers (3). The causes of female infertility are varied, including physiological, psychological, behavioral, and genetic factors (4). The process of diagnosing female infertility is therefore complex and time-consuming (5). The first step is to evaluate the medical history of the patient and to perform a physical examination, followed by laboratory tests (6). The function of the ovaries, fallopian tubes, and uterus is then evaluated, and the clinician finally makes a diagnosis by combining all of this information and relying on his or her experience (7). It takes at least 1–2 years from trying to conceive for about 1 year to confirm the diagnosis of infertility in the hospital. Therefore, early diagnosis of the condition is essential to shorten the time to successful conception. While imaging plays an indispensable role in assessing anatomic abnormalities, tubal obstruction, and ovarian reserve, it is not suitable for large-scale screening (8). Moreover, the diagnosis and treatment of infertility are complicated by various factors that must be considered (9). Therefore, there is a need to establish a simpler clinical screening index to be used for early prevention and intervention in cases of female infertility.

Pregnancy loss is defined as the natural termination of a pregnancy prior to fetal viability, and it includes spontaneous, missed, and incomplete abortions, as well as a molar pregnancy (10). Pregnancy loss is a common problem among women of childbearing age, with a reported incidence of 10-30% out of all detected pregnancies (11). A failed pregnancy can occur at any stage of pregnancy for a variety of reasons and may be associated with key physiological changes in the embryo, the uterine environment, and the ovaries (12, 13). Women with a history of pregnancy loss have higher rates of psychological conditions and chronic diseases (14). Although most cases of miscarriage are sporadic, some couples experience repeated miscarriages, which is a challenging situation to be addressed clinically (15). The causes of sporadic and recurrent miscarriage are multiple, but the risk of adverse pregnancy outcomes in almost all cases is influenced by prior obstetric history (16). Ultrasound is critical in the assessment and management of pregnancies of unknown location and can be of help in the differential diagnosis of early miscarriage, pregnancy of unknown location, and ectopic pregnancy (17). However, it cannot be used to assess the causes of unexplained pregnancy loss. The diagnostic criteria, treatment, preventive measures, and prediction methods for recurrent pregnancy loss vary globally as there is no international consensus on a definition (11, 18). Although the effects on couples are well documented, pregnancy loss is an understudied disorder with no precise diagnostic model or obvious treatment. Therefore, it is important to develop a precise and simple model to predict pregnancy loss in advance.

In general, the risk of recurrence of sporadic early pregnancy loss is low (approximately 12% to 14%) (15). However, pregnancy outcomes affect the proportion of recurrent miscarriages (19). Women affected by pregnancy loss have a 60% to 70% chance of a successful pregnancy; therefore, pregnancy loss is not the same as infertility (20). However, how to distinguish between these two conditions has rarely been addressed in the literature.

The current application of machine learning (ML) in healthcare highlights the potential to enhance disease diagnosis and clinical care, thus achieving early warning, improving patient outcomes, and increasing the diagnostic efficiency of clinicians (21, 22). The detection of laboratory indicators has made a significant contribution to disease diagnosis, and their synergy with ML algorithms can provide superior diagnostic accuracy and reduce false positives (23). However, its adoption in clinical practice for the diagnosis of infertility and pregnancy loss has not yet been realized, and the evaluation of ML-based diagnostic technologies in terms of infertility and pregnancy loss outcomes remains an ongoing endeavor.

The current definition of infertility acknowledges the importance of the total amount of time during which the patient has sought to become pregnant and the negative impact of age. To reduce the time to intervention and improve prognosis, the present study aimed to establish a simple and efficient method that provides early warning of infertility and potential pregnancy loss. We also systematically investigated the effect of 25-hydroxy vitamin D3 (25OHVD3) on infertility and pregnancy loss and whether the combination of 25OHVD3 and other clinical indicators can aid in the diagnosis of these conditions.

## 2 Materials and methods

### 2.1 Sample collection

**Figure 1:**
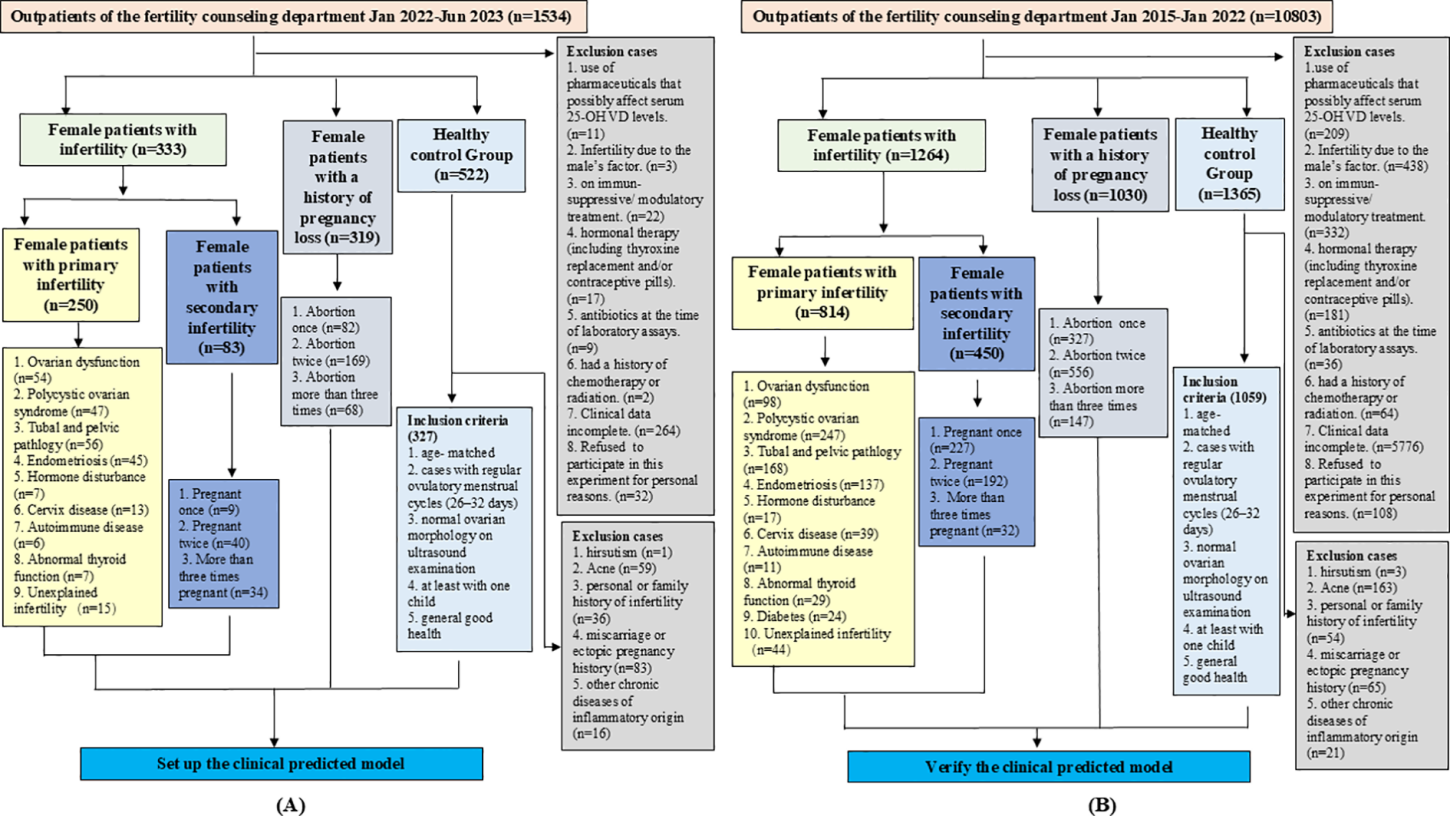
Patient inclusion and exclusion criteria, and flowchart for recruitment. **(A)** Flowchart for recruiting patients to establish diagnostic models. **(B)** Flowchart for recruiting patients to verify the diagnostic models.

In this study, we collected data from female patients who visited Xijing Hospital (Xi’an, China) from January 1, 2022, to June 1, 2023. All patients underwent medical history evaluation and physical examination, as well as clinical laboratory and ultrasound tests. All the included patients were diagnosed by gynecologists and infertility specialists and had a clear diagnosis that followed the appropriate guidelines (24–26). The participants were divided into two groups (333 patients diagnosed with infertility and 319 patients diagnosed with failed pregnancy). In addition, a third group (control) of 327 age-matched healthy women was included in the study. The first group of patients included cases of infertility related to conditions in the fallopian tubes, cervix, uterus, and ovaries, as well as cases in which the cause of infertility was unknown. The second group included patients with a history of abortion or ectopic pregnancy but who had not been diagnosed with infertility. The inclusion and exclusion criteria, as well as a flow chart for patient recruitment, are shown in **Figure 1**. To assess the validity of our model, we also collected data from 2,294 female patients treated at the Fertility and Infertility Center of Xijing Hospital from January 1, 2015, to January 1, 2022, with a clear diagnosis of infertility (1264) and pregnancy loss (1030), as well as from 1059 age-matched healthy women. The research protocol was approved by the Ethics Committee of Xijing Hospital (KY20212027-C-1), and informed consent was obtained via telephone interviews because of the retrospective nature of the study.

### 2.2 Data collection

Serum levels of 25OHVD3 and 25-hydroxy vitamin D2 (25OHVD2) were analyzed using high performance liquid chromatography-mass spectrometry (HPLC-MS/MS). All the laboratory tests were analyzed by the Clinical Laboratory Department of Xijing Hospital, and the results were stored in the Laboratory Information System (LIS). All consultation and basic information were obtained from the Hospital Information System, LIS, and follow-up telephone interviews. All the data were collected by more than three independent technicians and statisticians in accordance with the standardization requirements. All electronic data involved in this study were stored on independent USB drives. The USB flash drive and paper document information were stored in a confidential cabinet, which had dual locks and the keys were independently kept by two fixed individuals. USB flash drives could only be used on designated, protected, and confidential computers. The confidential cabinet and confidential computer were managed and protected by dedicated personnel to ensure that the data and information of participants were not leaked. The data included basic patient information, demographic information, physical examination results, diagnosis, infertility period, smoking status, alcohol consumption, and other information (**Table 1**).

**Table 1:** Patient characteristics.

Variables	Female controls (n=327)	Female patients with pregnancy loss (n=319)	Female patients with infertility (n=333)
Body Mass Index (kg/m^2^)	24.75 ± 4.12	25.37 ± 3.61	24.13 ± 3.26
Smoking History (%)	3.77	6.06	6.73
Alcohol History (%)	1.89	3.13	3.00
Diabetic (%)	0.00	6.06	5.77
Hypertension (%)	0.00	6.67	11.54
Regular Sporting (%)	7.55	27.27	9.62

### 2.3 Sample pretreatment for 25OHVD2 and 25OHVD3 detection

For HPLC-MS/MS detection of 25OHVD2 and 25OHVD3, 500 μL of internal standard solution was added to 100 μL of serum, following which the homogeneous solution was shaken and mixed for 1 min and centrifuged at 15,000 rpm for 10 min. The supernatant was then transferred for N2 drying. For the derivatization reaction, a 4-phenyl-1,2,4-triazoline-3,5-dione solution was added to the drying sample and incubated at 25°C for 30 min. The derivatization solution was subjected to N2 drying, following which 50 µL of methanol was added, mixed for 1 min, and centrifuged at 15,000 rpm for 10 min at 25°C. The supernatant was prepared for HPLC-MS/MS detection.

### 2.4 HPLC-MS/MS analysis of 25OHVD2 and 25OHVD3

In this study, 25OHVD2 and 25OHVD3 levels were analyzed using an HPLC-MS/MS system equipped with an Agilent 1200 HPLC system (Agilent1200, Waldbronn, Germany) and an API 3200 QTRAP MS/MS system (Sciex, Darmstadt, Germany). Mobile phase A consisted of an aqueous solution containing 1% formic acid and 1% ammonium formate, while phase B consisted of a methanol solution containing 1% formic acid and 1% ammonium formate. The optimized gradient elution was operated at a flow rate of 0.6 ml/min: 0 to 0.1 min, 70% B; 0.1 to 0.6 min, 70–95% B; 0.6 to 3.1 min, 95% B; and 3.1 to 4.0 min, 95–70% B. An autosampler was set to inject 20 μL at each step. MS data were detected via electrospray ionization (ESI) in a positive ion mode, and the remaining parameters were as follows: multiple reaction monitor scan type; collection ion pair: 25(OH)VD_2_: 619.3/298.3, d3-25(OH)VD_2_: 622.3/301.3, 25(OH)VD_3_: 607.3/298.3, d3-25(OH)VD_3_: 610.3/301.3; ion spray voltage, 5.5kV; ion source temperature, 600°C; curtain gas (CUR), 40.0 psi; nebulizer gas (GS1), 55.0 psi; declustering potential (DP), 40 V; entrance potential (EP), 4.0 V; collision energy (CE), 27; and collision cell exit potential (CXP), 3.0.

### 2.5 Feature selection and establishment of the diagnostic models

100+ clinical indicators were listed in **Supplementary Table S1**. All the missing values were supplemented by mean values. All data had not been normalized. Spearman correlation, recursive feature elimination (REF), and mutual information (MI)) were selected as methods of feature selection for the model of diagnosis. Spearman correlation analysis is used to assess the monotonic relationship between two continuous or ordered variables (27). It is used to characterize the correlation between two variables that have ordinal or distributional characteristics that cannot be described in terms of mean and standard deviation. MI is a metric that quantifies the dependence and relationship between two variables and represents the amount of information provided by one probabilistic variable about the other (28). RFE is the main representative of wrap-around feature selection, which brings classification algorithms into the process of feature selection to eliminate redundancy between features and output the best combination of features (29). To establish effective diagnostic models, thirty indicators with the highest contribution values were screened and cross-validated using these three methods. Common indicators were used to build the model. The selected features will vary with the number of samples and different screening methods, so we use multiple ML algorithms and metrics to simultaneously establish, validate, and evaluate our diagnostic model. Gaussian naive bayes (GNB), K-nearest neighbors (KNN), decision tree (DT), logistic regression (LR), and eXtreme gradient boosting (XGBoost) were used to develop and evaluate diagnostic models based on the common indicators. The performance of our model was improved by using a ten-fold crossover (train: 9, test:1, random allocation). External datasets were independently validated to enhance the generalizability of the models.

### 2.6 Statistical analysis

SPSS 23.0 (IBM, Armonk, NY, USA) was used for data analysis. Quantitative data are expressed as mean ± standard deviation. Group comparisons were made using the chi-square test for categorical variables and one-way analysis of variance (ANOVA), and the Kruskal-Wallis test for continuous variables. The multiple comparisons using the Bonferroni correction to reduce the risk of Type I error, and statistical significance was set at p<0.05/N, where N represents the number of comparisons. R Software (version 3.6.2, R Statistical Computing Project) was used for data visualization. The Python language was used for indicator screening and for diagnostic model building and evaluation. Orthogonal partial least squares discriminant analysis (OPLS-DA) was performed to determine overall differences between the groups. Risk factors for infertility and pregnancy loss were evaluated using logistic regression analysis. The diagnostic performance of the model was analyzed using receiver operating characteristic (ROC) curves.

## 3 Results

### 3.1 Patient characteristics

As shown in **Figure 1**, a total of 1534 patients were initially included in this study for modeling, and 360 were initially excluded according to the exclusion criteria. A total of 522 women were initially included in the healthy control group, with 195 excluded according to the telephone follow-up results, and the remaining 327 were finally enrolled. The average ages of patients with infertility (n=333), those with pregnancy loss (n=319), and healthy individuals (n=327) were 30.40 ± 14.72, 30.71 ± 14.10, and 31.15 ± 14.83 years, respectively, with no significant differences among the groups. In the infertility group, 75.08% of the patients had primary infertility and 24.92% had secondary infertility. In the secondary infertility group, 10.85% of the patients had been pregnant once, 48.19% had been pregnant twice, and 40.96% had been pregnant three times or more. In the pregnancy loss group, 25.71% of the patients had experienced an abortion once, 52.98% had experienced it twice, and 21.31% had experienced it three times or more. The main characteristics of each group are presented in **Figure 1A** and **Table 1**. Drinking and smoking were more common in the infertility group compared to the control group. The clinical indicators of each group are also presented in **Supplementary Table S1**. The basic information, inclusion and exclusion criteria for the 2294 patients and 1059 healthy individuals included in the validation set were also shown in **Figure 1B**.

### 3.2 Distribution of measured indicators in each group

As shown in **Supplementary Figure S1A**, the overall difference in 100+ clinical indicators clearly distinguished the infertility group from the normal control group. Among female patients, significant differences in the levels of the following indicators were observed between the group of patients with infertility and the control group: 25OHVD2, 25OHVD3, prothrombin time (PT), luteinizing hormone (LH), erythrocyte sedimentation rate (ESR), hyaline cast (Hy. CAST), high-density lipoprotein (HDL-C), thrombin time (TT), anticardiolipin antibodies (ACA), creatinine (CRE), homocysteine (HCY), mucous strands (MUCUS), NonSEC, mean corpuscular hemoglobin concentration (MCHC), triglyceride (TG), progesterone (PROG), estradiol (E2), urea (BUN), urinary epithelial cells (EC), gamma-glutamyl transferase (GGT), aspartate aminotransferase (AST), red blood cells (RBC), cystatin C (CysC), and pathocast (Path CAST) (VIP>1.00; **Supplementary Figure S1B**). Notably, 25OHVD3 was the indicator exhibiting the biggest differences between these two groups.

Similarly, an overall difference of 100+ clinical markers clearly distinguished the pregnancy loss group from the normal control group (**Supplementary Figure S1C**). Fourteen clinical indicators, including anti-thyroid peroxidase antibody (TPOAb), monocytes (MONO), neutrophilic granulocytes (NEUT), eosinophils (EO), human papillomavirus 59 (HPV59), red blood cell distribution width (RDW), Path CAST, red blood cell specific volume (HCT), free thyroxine 4 (FT4), human papillomavirus 81 (HPV 81), urine potential of hydrogen (UPH), albumin (ALB), basophils (BASO), and alanine aminotransferase (ALT), were significantly different between the group of patients with pregnancy loss and the control group (**Supplementary Figure S1D**).

### 3.3 Distribution of 25OHVD levels in each group

**Figure 2:**
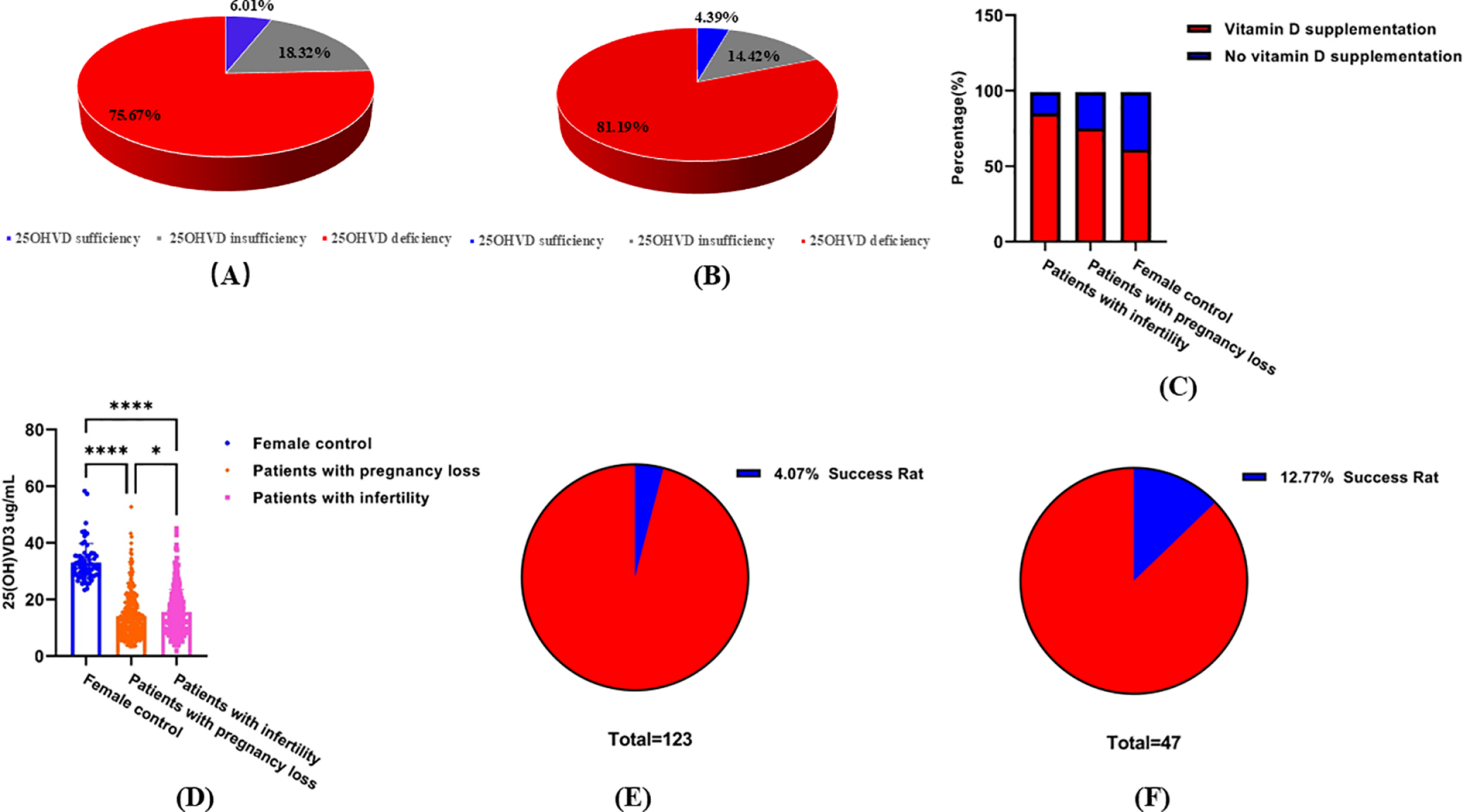
Serum 25OHVD3 levels. **(A)** Deficiency, insufficiency, and sufficiency percentage in 25OHVD of the patients with infertility. **(B)** Deficiency, insufficiency, and sufficiency percentage in 25OHVD of the patients with pregnancy loss. **(C)** supplementation of female groups. **(D)** Vitamin D concentration in different study groups. *, p < 0.05; ****, p < 0.0001. **(E)** successful pregnancy rate of patients with infertility (25OHVD3<20ng/mL) choosing *in vitro* fertilization technology. **(F)** successful pregnancy rate of patients with infertility (25OHVD3>20ng/mL) choosing *in vitro* fertilization technology.

Levels above 30, 20–30, and below 20 ng/ml are regarded as normal, inadequate, and deficient, respectively (30). The study participants were divided into three groups according to their 25OHVD levels. The percentage of patients included in each group is shown in **Figure 2**. Deficiency, insufficiency, and sufficiency in 25OHVD were observed in 75.68%, 18.32%, and 6.01% of the patients with pregnancy loss, respectively (**Figure 2A**). Among the patients with infertility, the rates of 25OHVD deficiency, insufficiency, and sufficiency were 81.19%, 14.42%, and 4.39%, respectively (**Figure 2B**). Remarkably, we found that although 85% of the patients with infertility, 75% of the patients with pregnancy loss, and 61% of the healthy women in the control group had been supplemented with vitamin D (**Figure 2C**), the concentration of 25OHVD3 analyzed using ANOVA in the first two groups was much lower than that observed in healthy individuals (**Figure 2D**). Although we did not find a dose-response relationship between vitamin D level categories and infertility risk, our results showed that 48.84% of patients with infertility (25OHVD3<20ng/mL) chose *in vitro* fertilization technology, but only 4.07% of the patients successfully became pregnant (**Figure 2E**). In contrast, 57.32% of patients with infertility (25OHVD3>20ng/mL) chose *in vitro* fertilization technology, but 12.77% of the patients successfully became pregnant (**Figure 2F**).

### 3.4 Model development and diagnostic performance

**Figure 3:**
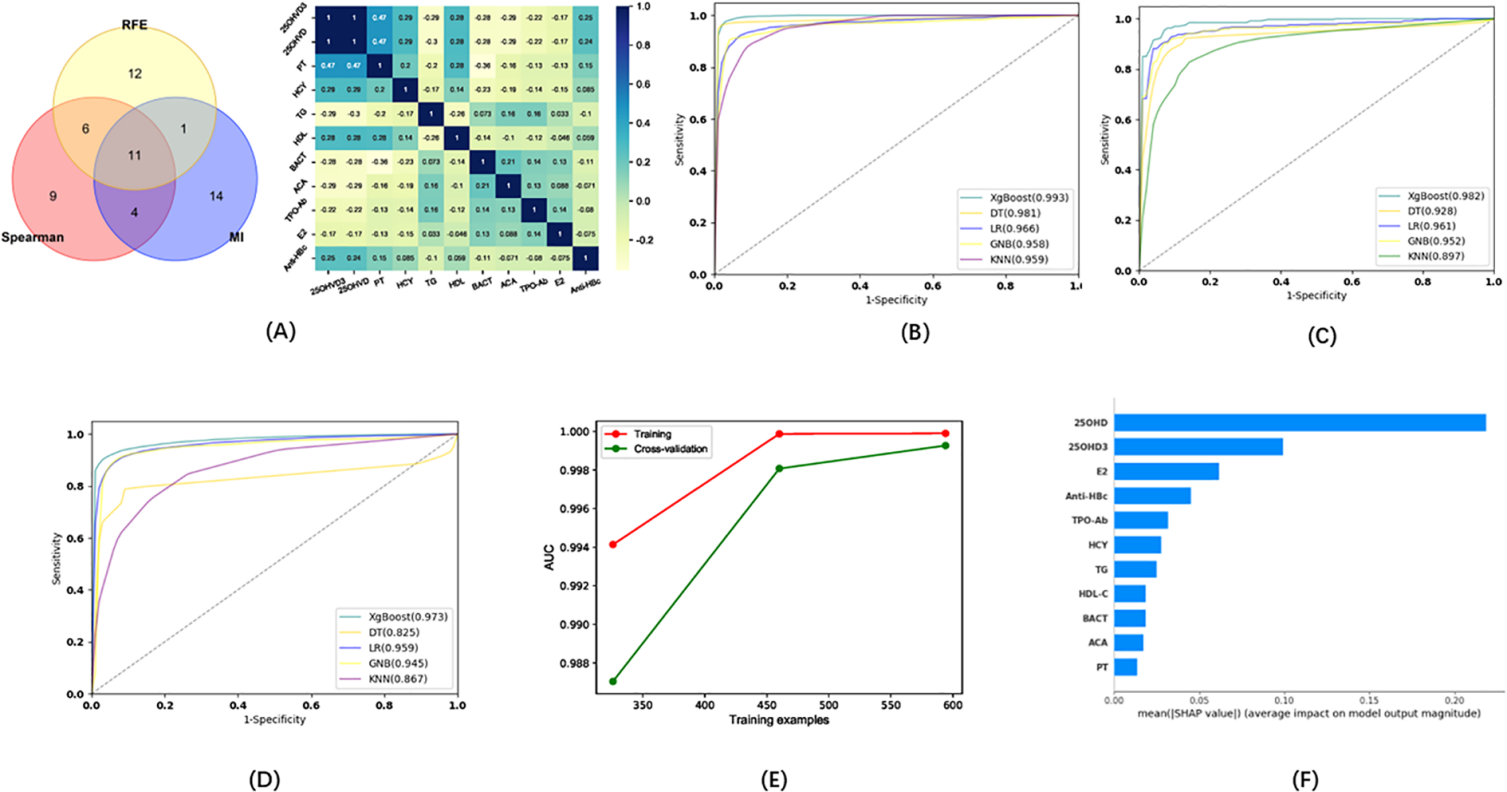
**(A)** Wayne diagrams for the MI, REF, and Spearman methods used to screen candidates for differentiating patients with infertility from healthy individuals. Hotspot map of candidate indicators for this differentiation. **(B)** Receiver operating characteristic (ROC) curve for the training set in the model for infertility diagnosis. **(C)** ROC curve for the test set in the model for infertility diagnosis. **(D)** ROC curve for the validation set in the model for infertility diagnosis. **(E)** Learning curve for the training set in the model for infertility diagnosis. **(F)** SHAP model for 11 indicators of the model for infertility diagnosis.

Eleven indicators were selected via three methods (Spearman, REF, MI) as candidates for the model of infertility diagnosis: High-density lipoprotein (HDL), TG, 25OHVD3, PT, ACA, 25OHVD, HCY, urine bacterial count (BACT), TPOAb, E2, and hepatitis B core antibody (Anti-HBc) (**Figure 3A**). Five ML algorithms were used to establish and evaluate the model based on these 11 indicators. The results showed that the sensitivity for the training set was higher than 86.52%, the specificity was higher than 91.23%, the accuracy was higher than 89.70%, and the area under the curve (AUC) of the ROC was higher than 0.958 (**Figure 3B**; **Table 2**. The sensitivity for the testing set was higher than 81.81%, the specificity was higher than 88.08%, the accuracy was higher than 84.70%, and the AUC of the ROC was higher than 0.928 (**Figure 3C**; **Table 2**). The sensitivity for the validation set was higher than 74.81%, the specificity was higher than 84.00%, the accuracy was higher than 79.00%, and the AUC of the ROC was higher than 0.825 (**Figure 3D**; **Table 2**). XGboost performed the best by comparing the results of training sets, test sets, and validation sets in these five ML. Learning curve could illustrate the impact of the number of training samples on the model performance (**Figure 3E**). The results indicated that the ML algorithm used in this study did not exhibit overfitting or underfitting. The model has basically reached the performance platform and does not require additional data for further training. The SHAP model of the eleven indicators in the diagnostic model revealed that 25OHVD and 25OHVD3 contributed the most, followed by E2, Anti-HBc, TPOAb, HCY, TG, HDL, BACT, ACA, and PT (**Figure 3F**).

**Table 2:** Evaluation of the model for infertility and control, pregnancy loss and control, infertility and pregnancy loss.

Model	Algorithms	Set	Sensitivity	Specificity	Accuracy	Area under the curve
Infertility and Control	XgBoost	Train	96.60% (95.19%-98.01%)	94.41% (92.60%-96.22%)	92.33% (90.20%-94.46%)	0.993 (0.987-0.999)
		Test	92.33% (90.20%-94.46%)	95.84% (94.28%-97.40%)	94.09% (92.22%-95.96%)	0.982 (0.972-0.992)
		Verify	90.22% (88.97%-91.47%)	96.92% (96.23%-97.61%)	93.28% (92.25%-94.31%)	0.973 (0.967-0.979)
	DT	Train	96.03% (94.50%-97.56%)	98.54% (97.62%-99.46%)	97.27% (96.01%-98.53%)	0.981 (0.970-0.992)
		Test	91.07% (88.77%-93.37%)	94.01% (92.13%-95.89%)	92.27% (90.13%-94.41%)	0.928 (0.907-0.949)
		Verify	79.04% (77.23%-80.85%)	89.89% (88.62%-91.16%)	83.99% (82.40%-85.58%)	0.825 (0.808-0.842)
	Knn	Train	88.18% (85.54%-90.82%)	91.23% (88.96%-93.50%)	89.70% (87.24%-92.16%)	0.959 (0.943-0.975)
		Test	81.81% (78.58%-85.04%)	88.08% (85.43%-90.73%)	84.70% (81.72%-87.68%)	0.987 (0.872-0.922)
		Verify	74.81% (72.85%-76.77%)	84.00% (82.41%-85.59%)	79.00% (77.19%-80.81%)	0.867 (0.852-0.882)
	LR	Train	90.36% (87.98%-92.74%)	94.32% (92.49%-96.15%)	92.32% (90.19%-94.45%)	0.966 (0.952-0.980)
		Test	89.93% (87.49%-92.37%)	93.44% (91.47%-95.41%)	91.52% (89.28%-93.76%)	0.961 (0.946-0.976)
		Verify	89.66% (88.38%-90.94%)	93.27% (92.24%-94.30%)	91.30% (90.12%-92.48%)	0.959 (0.951-0.967)
	GNB	Train	86.52% (83.71%-89.33%)	96.94% (95.60%-98.28%)	91.68% (89.46%-93.90%)	0.958 (0.942-0.974)
		Test	86.24% (83.40%-89.08%)	96.68% (95.28%-98.08%)	91.36% (89.10%-93.62%)	0.952 (0.935-0.969)
		Verify	83.81% (82.21%-85.41%)	96.38% (95.63%-97.13%)	89.54% (88.25%-90.83%)	0.945 (0.936-0.954)
Pregnancy Loss and Control	XgBoost	Train	96.45% (94.98%-97.92%)	98.20% (97.15%-99.25%)	97.33% (96.05%-98.61%)	0.993 (0.986-1.000)
		Test	94.42% (92.57%-96.27%)	96.83% (95.44%-98.22%)	95.81% (94.21%-97.41%)	0.987 (0.978-0.996)
		Verify	94.02% (92.96%-95.08%)	95.13% (94.17%-96.09%)	94.58% (93.57%-95.59%)	0.973 (0.966-0.980)
	DT	Train	97.04% (95.70%-98.38%)	99.29% (98.63%-99.95%)	98.18% (97.13%-99.23%)	0.985 (0.975-0.995)
		Test	94.53% (92.70%-96.36%)	96.62% (95.18%-98.06%)	95.66% (94.03%-97.29%)	0.959 (0.943-0.975)
		Verify	94.51% (93.49%-95.53%)	86.88% (85.31%-88.45%)	90.65% (89.32%-91.98%)	0.900 (0.886-0.914)
	Knn	Train	93.34% (91.32%-95.36%)	95.31% (93.62%-97.00%)	94.34% (92.48%-96.20%)	0.987 (0.978-0.996)
		Test	90.78% (88.41%-93.15%)	94.95% (93.19%-96.71%)	92.88% (90.79%-94.97%)	0.964 (0.949-0.979)
		Verify	87.25% (85.70%-88.80%)	94.65% (93.64%-95.66%)	91.00% (89.70%-92.30%)	0.948 (0.938-0.958)
	LR	Train	92.02% (89.81%-94.23%)	96.94% (95.57%-98.31%)	94.51% (92.68%-96.34%)	0.972 (0.959-0.985)
		Test	98.55% (97.61%-99.49%)	88.02% (85.32%-90.72%)	96.89% (95.51%-98.27%)	0.966 (0.952-0.980)
		Verify	91.15% (89.86%-92.44%)	96.14% (95.29%-96.99%)	93.96% (92.89%-95.03%)	0.931 (0.920-0.942)
	GNB	Train	93.87% (91.93%-95.81%)	95.18% (93.46%-96.90%)	94.53% (92.70%-96.36%)	0.977 (0.965-0.989)
		Test	93.73% (91.77%-95.69%)	94.90% (93.13%-96.67%)	94.43% (92.58%-96.28%)	0.973 (0.960-0.986)
		Verify	90.68% (89.35%-92.01%)	93.05% (91.90%-94.20%)	91.88% (90.64%-93.12%)	0.959 (0.950-0.968)
Infertility and Pregnancy Loss	XgBoost	Train	88.58% (85.98%-91.18%)	91.91% (89.72%-94.10%)	90.22% (87.81%-92.63%)	0.956(0.940-0.972)
		Test	84.85% (81.87%-87.83%)	86.09% (83.23%-88.95%)	85.28% (82.34%-88.22%)	0.925(0.904-0.946)
		Verify	85.32% (83.79%-86.85%)	85.51% (83.99%-87.03%)	85.41% (83.88%-86.94%)	0.928 (0.917-0.939)
	DT	Train	77.05% (73.46%-80.64%)	91.52% (89.28%-93.76%)	84.12% (81.08%-87.16%)	0.903(0.879-0.927)
		Test	69.74% (65.75%-73.73%)	86.38% (83.55%-89.21%)	77.93% (74.40%-81.46%)	0.810(0.777-0.843)
		Verify	69.30% (67.18%-71.42%)	82.63% (80.97%-84.29%)	75.29% (73.34%-77.24%)	0.795 (0.917-0.939)
	Knn	Train	70.83% (66.89%-74.77%)	91.11% (88.82%-93.40%)	80.76% (77.44%-84.08%)	0.905(0.881-0.929)
		Test	58.74% (54.39%-63.09%)	83.19% (80.06%-86.32%)	70.56% (66.61%-74.51%)	0.767(0.731-0.803)
		Verify	55.14% (52.79%-57.49%)	83.71% (82.10%-85.32%)	67.97% (65.81%-70.13%)	0.761 (0.742-0.780)
	LR	Train	74.93% (71.21%-78.65%)	80.6% (77.26%-83.94%)	77.71% (74.17%-81.25%)	0.846 (0.816-0.876)
		Test	73.90% (70.12%-77.68%)	80.10% (76.73%-83.47%)	76.85% (73.25%-80.45%)	0.838 (0.807-0.869)
		Verify	76.05% (74.12%-77.98%)	79.92% (78.14%-81.70%)	77.79% (75.93%-79.65%)	0.852 (0.837-0.867)
	GNB	Train	54.05% (49.63%-58.47%)	96.86% (95.50%-98.22%)	75% (71.28%-78.72%)	0.806 (0.773-0.839)
		Test	52.58% (48.15%-57.01%)	96.02% (94.49%-97.55%)	73.77% (69.98%-77.56%)	0.798(0.764-0.832)
		Verify	52.93% (50.56%-55.30%)	91.99% (90.86%-93.12%)	70.47% (68.38%-72.56%)	0.796 (0.778-0.814)

**Figure 4:**
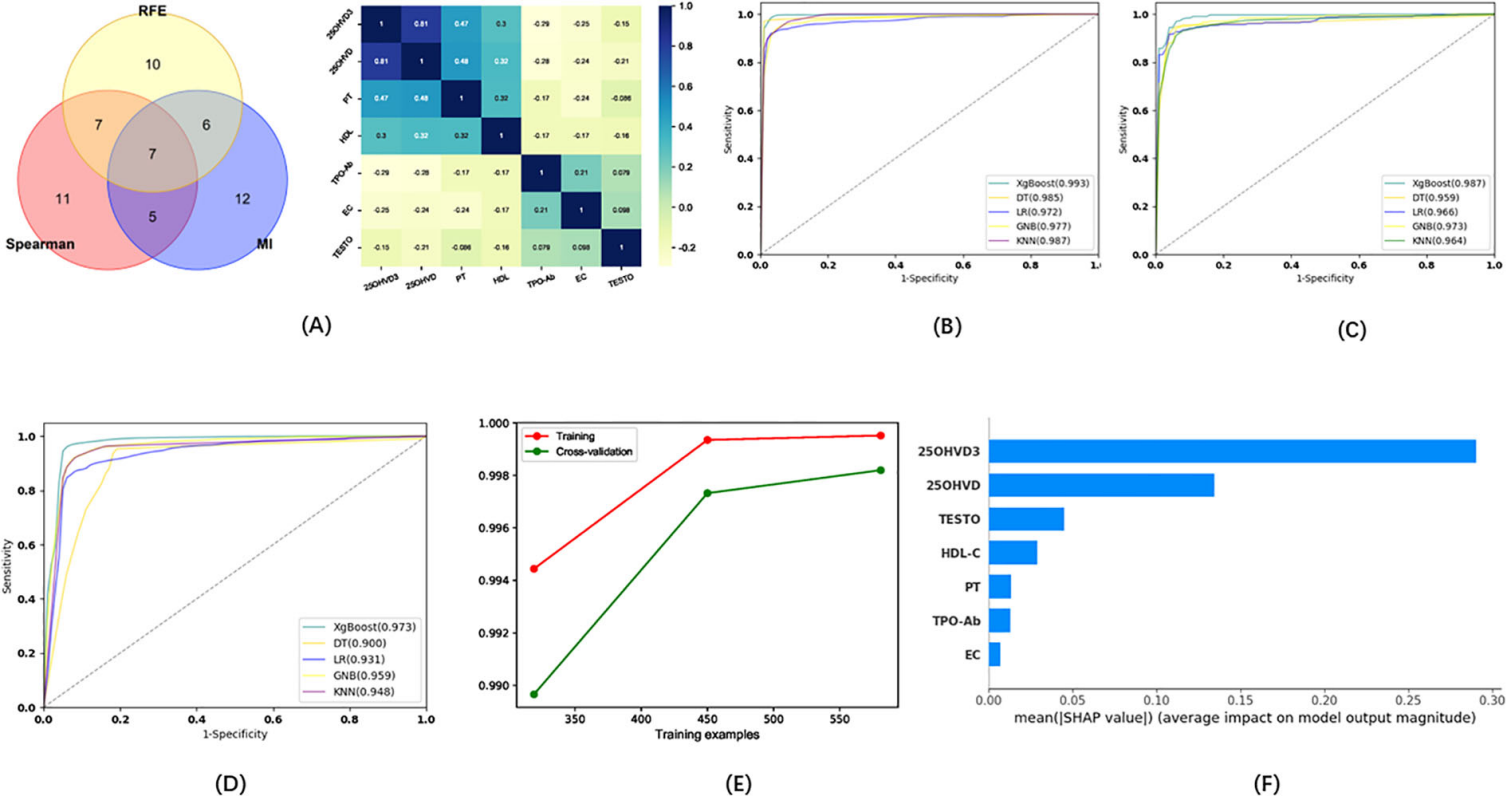
**(A)** Wayne diagrams for MI, REF, and SPEARSON methods used to screen candidates for differentiating patients with pregnancy loss from healthy individuals. Hotspot map of candidate indicators for this differentiation. **(B)** ROC curve for the training set in the model for the prediction of pregnancy loss. **(C)** ROC curve for the test set in the model for the prediction of pregnancy loss. **(D)** ROC curve for the validation set in the model for the prediction of pregnancy loss. **(E)** Learning curve for the training set in the model for the prediction of pregnancy loss. **(F)** SHAP model for 7 indicators of the model for the prediction of pregnancy loss.

Seven indicators were selected via the three methods as candidates for the model of pregnancy loss: EC, TPOAb, HDL, testosterone (TESTO), 25OHVD3, PT, and 25OHVD (**Figure 4A**). Five ML algorithms were used to evaluate the model based on these seven indicators. The results showed that the sensitivity for the training set was higher than 92.02%, the specificity was higher than 95.18%, the accuracy was higher than 94.34%, and the AUC of the ROC curve was higher than 0.972 (**Figure 4B**; **Table 2**). The sensitivity for the testing set was higher than 90.78%, the specificity was higher than 88.02%, the accuracy was higher than 92.88%, and the AUC of the ROC curve was higher than 0.948 (**Figure 4C**; **Table 2**). The sensitivity for the validation set was higher than 87.25%, the specificity was higher than 86.88%, the accuracy was higher than 90.65%, and the AUC of the ROC was higher than 0.900 (**Figure 4D**; **Table 2**). XGboost performed the best by comparing the results of training sets, test sets, and validation sets in these five ML algorithms. The results of the learning curve indicated that the ML algorithm used in this study did not exhibit overfitting or underfitting (**Figure 4E**). The model had basically reached the performance platform and did not require additional data for further training. The SHAP model of the 7 indicators in the diagnostic model revealed that 25OHVD3 and 25OHVD contributed the most, followed by TESTO, HDL, PT, TPOAb, and EC (**Figure 4F**).

**Figure 5:**
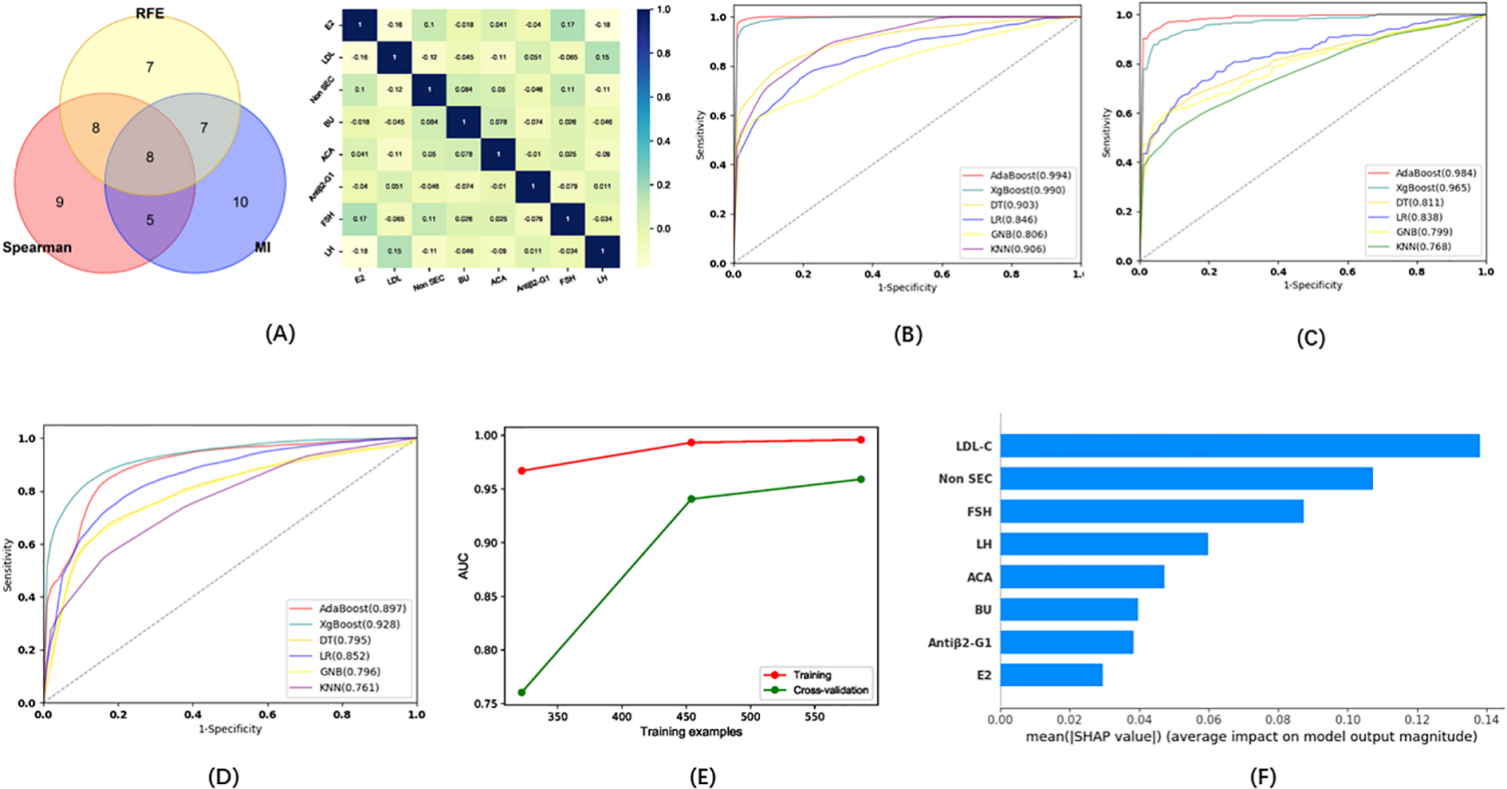
**(A)** Wayne diagrams for the MI, REF, and SPEARSON methods used to screen candidates for differentiating patients with infertility from those with pregnancy loss. Hotspot map of candidate indicators for this differentiation. **(B)**. ROC curves for the training set in the model for the differentiation of patients with infertility and with pregnancy loss. **(C)** ROC curve for the test set in the model for the differentiation of patients with infertility and with pregnancy loss. **(D)** ROC curve for the validation set in the model for the differentiation of patients with infertility and with pregnancy loss. **(E)** Learning curve for the training set in the model for the differentiation of patients with infertility and with pregnancy loss. **(F)** SHAP model for 8 indicators of the model for the differentiation of patients with infertility and with pregnancy loss.

In addition, we tried to develop a model capable of distinguishing between patients with infertility and those with predicted pregnancy loss. Eight indicators (E2, LDL, Non SEC, BU, ACA, Antiβ2-G1, FSH, LH) were selected via the three methods as candidates for this model (**Figure 5A**). Five ML algorithms were used to evaluate the diagnostic model based on these eight indicators. The results showed that the sensitivity for the training set was higher than 54.05%, the specificity was higher than 80.60%, the accuracy was higher than 75.00%, and the AUC of the ROC was higher than 0.767 (**Figure 5B**; **Table 2**). The sensitivity for the testing set was higher than 52.58%, the specificity was higher than 80.10%, the accuracy was higher than 70.56%, and the AUC of the ROC was higher than 0.806 (**Figure 5C**; **Table 2**). The sensitivity for the validation set was higher than 52.93%, the specificity was higher than 79.92%, the accuracy was higher than 67.97%, and the AUC of the ROC was higher than 0.761 (**Figure 5D**; **Table 2**). XGboost performed the best by comparing the results of training sets, test sets, and validation sets in these five ML algorithms. The results of the learning curve indicated that the ML algorithm used in this study did not exhibit overfitting or underfitting (**Figure 5E**). The model had basically reached the performance platform and did not require additional data for further training. The SHAP model of the eight indicators in the diagnostic model revealed that LDL and Non SEC contributed the most, followed by FSH, LH, ACA, BU, Antiβ2-G1, and E2 (**Figure 5F**).

### 3.5 Significantly different indicators in infertility risk factor assessment

To identify potential indicators of infertility risk, we used a binary logistic regression analysis to assess the relationship between these indicators and infertility. As shown in **Supplementary Table S2**, ESR60M, HDL, PT, 25OHVD3, LH, TT, CysC, ACA, HCY, CRE, MCHC, GGT, and MUCUS were statistically significant risk factors for female infertility.

**Figure 6:**
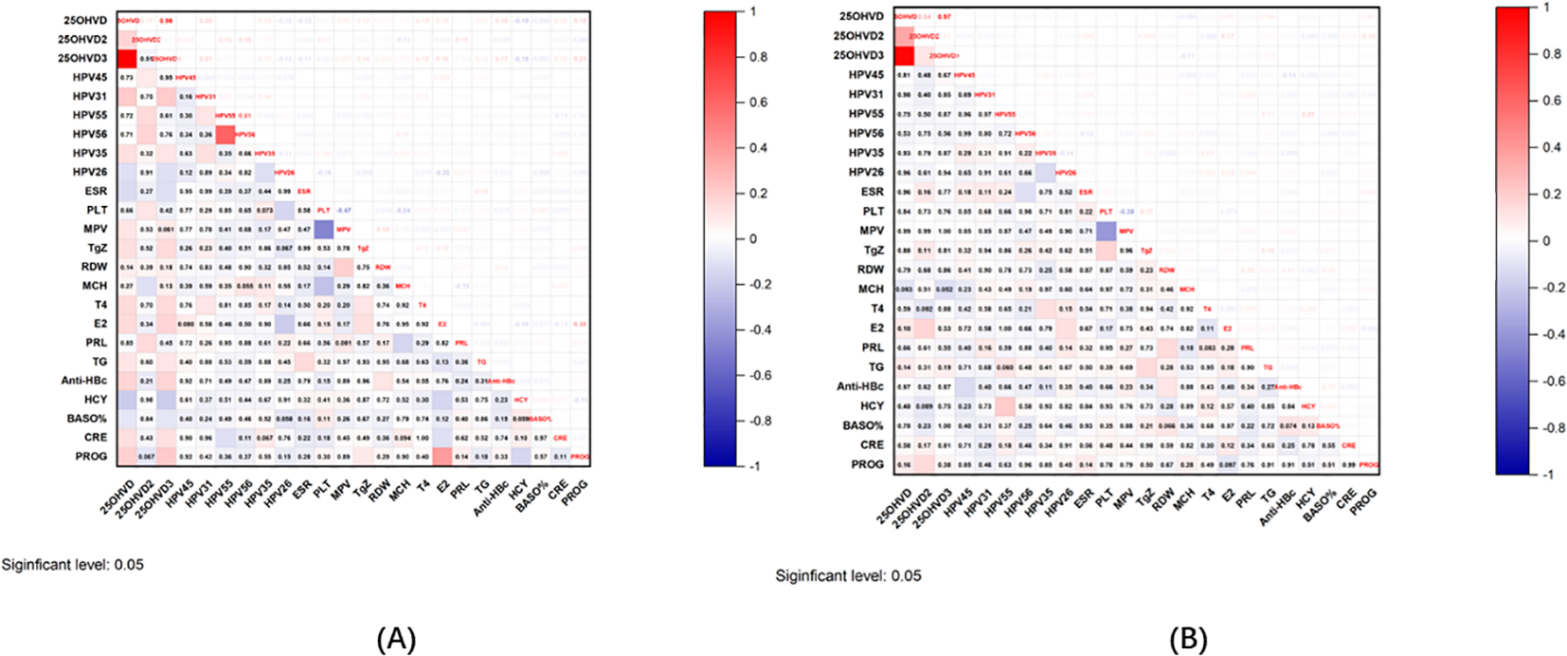
**(A)** Correlation graph of 25OHVD2, 25OHVD3, and 25OHVD in infertility with other clinical indicators. **(B)** Correlation graph of 25OHVD2, 25OHVD3, and 25OHVD in pregnancy loss with other clinical indicators.

25OHVD3 exhibited the most marked difference in cases of infertility. To investigate its role in the occurrence and development of infertility, we also looked at its correlation with a variety of clinical indicators. Intriguingly, 25OHVD3 was correlated with HPV31, HPV35, HPV26, ESR, thyroglobulin (TgZ), T4, E2, TG, Anti-HBc, HCY, BASO, CRE, and PROG (**Figure 6A**). 25OHVD2 was also correlated with HPV45, HPV55, HPV56, platelet (PLT), mean corpuscular hemoglobin (MCH), and prolactin (PRL) (**Figure 6A**). In patients with pregnancy loss, 25OHVD3 was not significantly associated with any of these clinical markers (**Figure 6B**). 25OHVD2 was significantly correlated with E2 and PROG (**Figure 6B**).

## 4 Discussion

Many couples expecting to become pregnant struggle with infertility, the risk of which is reported to be equal for male and female patients (31). The causes of infertility are multiple (32). Previous reports have indicated that the causes of infertility are unknown in approximately 30% of infertile couples (33). The age of the female partner is among the factors that have been associated with unexplained infertility (34). Given the multitude of factors that must be considered, developing a system that can aid in the efficient, early, and accurate diagnosis of infertility remains both necessary and clinically challenging (35).

Our analyses identified positive correlations between the levels of BUN, ACA, HCY, MCHC, GGT, EC, TG, E2, CEA, and AST and female infertility, while negative correlations were observed between those of 25OHVD3, ESR60M, HDL-C, PT, TT, LH, CysC, CRE, MUCUS, and globulin and female infertility. Our results further indicate that HPV infection, abnormal coagulation function, thyroid dysfunction, abnormal blood lipid metabolism, 25OHVD deficiency or insufficiency, anemia, and abnormal liver function were risk factors for miscarriage and infertility. Over the past decade, our understanding regarding the benefits of vitamin D has improved significantly, particularly with regard to its non-skeletal functions (36). The vitamin D receptor (VDR) is expressed in most organs, suggesting that the roles of vitamin D extend beyond its functions in regulating calcium homeostasis and bone health (37). Numerous studies have reported associations between poor vitamin D status and cancer, allergies, immune disorders, cardiovascular metabolic diseases, irritable bowel syndrome, autism, muscle function, and brain function (36, 38). Given the numerous reports on the effects of vitamin D on other systems in the body, recent research has also focused on its role in human fertility (39). Vitamin D (cholecalciferol) has no biological activity; it must be activated by 25-hydroxylation in the liver, which converts cholecalciferol to the main circulating metabolite, 25-hydroxyvitamin D (25OHVD) (40, 41). Renal 1a-hydroxylase then converts 25OHVD to an active metabolite, 1,25(OH)_2_D, which binds to and activates VDR (42). The best method for assessing vitamin D status is to measure the serum concentration of 25OHVD, as it has a longer cyclic half-life and higher serum concentration than 1,25(OH)_2_D (43). The role of immunity in infertility and miscarriage has been demonstrated (44). Vitamin D signaling can regulate a variety of immune responses by regulating the differentiation and cycle of T cells, B cells, neutrophils, DC cells and other immune cells (45, 46). In addition, the enzyme CYP27B1, which produces the vitamin D hormone form 1,25(OH)_2_D, are expressed throughout the immune system. Notably, CYP27B1 expression in immune cells is independent of calcium homeostatic inputs (46). The importance of 1,25(OH)_2_D signaling in the regulation of the immune system is further emphasized by the numerous signaling pathways that control CYP27B1 expression in various immune cell types (47). In addition, vitamin D activates autophagy in a variety of cell types, including keratinocytes, hepatocytes, and endothelial cells, in response to cellular injury and oxidative stress (48). Therefore, we propose the hypothesis that vitamin D signaling could affect infertility and miscarriage by modulating immunity to influence the number of mature oocytes and the rate of blastocyst formation. In addition, Kinuta et al. found that VDR deficient mutant mice exhibited significant gonadal dysfunction, leading to high gonadotropin-induced hypogonadism and decreased ovarian aromatase activity (49). Therefore, VD is an important factor for the complete function of the gonads. While research regarding the relationship between vitamin D and fertility has yielded promising results, vitamin D deficiency has been associated with numerous diseases, meaning that its specificity for disease diagnosis remains poor (50). However, to the best of our knowledge, few studies have focused on whether the combination of 25OHVD and other clinical indicators can be useful in the diagnosis of infertility.

25OHVD3 was not only one of the indicators that showed the most marked difference in cases of infertility, but was also identified by all three of the methods used. To investigate its role in the occurrence and development of infertility, we also looked at its correlation with a variety of clinical indicators. Intriguingly, 25OHVD3 is associated with blood lipids, hormones, thyroid function, HPV infection, hepatitis B infection, sedimentation rate, renal function, coagulation function, and amino acids. However, in patients with pregnancy loss, although 25OHVD3 was also one of the most prominent indicators and was also identified by all three of the methods used, the correlation with HPV infection, coagulation function, platelet, thyroid function and other indicators disappeared. These results suggest that 25OHVD3 has a unique role in infertility, and its pathogenesis remains to be studied. The pathogenesis of infertility and that of pregnancy loss are indeed different, but the nature of the differences needs to be further studied.

Some authors have also attempted to develop new diagnostic methods for infertility. Cheng et al. established a cardiometabolic index (CMI) for diagnosing infertility (AUC=0.60, 95%CI: 0.56-0.65); the improved CMI index combined with BMI had a better predictive effect on infertility (AUC=0.722, 95%CI: 0.676-0.767) (51). Jiang et al. studied the plasma exosomes of 75 patients with polycystic ovary syndrome (PCOS) and used miR-126-3p, miR-146a-5p, miR-20b-5p, miR-106a-5p, and miR-18a-3p to distinguish PCOS patients from control individuals. The AUC of the ROC curve was 0.781 (52). However, there is still room for improvement in terms of infertility diagnosis. Since our results demonstrate that 25OHVD3 plays a role in the development of infertility, diagnosis based on multiple factors may have greater clinical significance. Similarly, we observed excellent diagnostic performance for the eleven factors included in our model for the diagnosis of female infertility, with AUC, sensitivity, and specificity values higher than 0.958, 86.52%, and 91.23%, respectively. In addition, we developed a diagnostic model to distinguish between infertility and pregnancy loss. Although the sensitivity of the models established by GNB, KNN, and DT ML in the verification set is not high (52.93%, 55.14%, and 69.30%, respectively), however the sensitivity of the models established by LR, and XgBoost ML in the verification set is 76.05%, and 85.32%, respectively, indicating that the models established by LR, and XgBoost, ML can be used to distinguish fertility from pregnancy loss. To the best of our knowledge, there are currently few diagnostic models for distinguishing infertility from pregnancy loss, and our results can fill in this gap. Moreover, the sensitivity and specificity of our model were markedly higher than those estimated for routine parameters and for most models that have been reported so far. The models we have developed are relatively simple, as the data can be obtained via routine laboratory analyses. Moreover, the present results may aid in the development of new indices for the diagnosis, treatment, and prevention of infertility. In addition, the models are suitable for use in large-scale screening to provide early warning of infertility, which can help ensure that patients do not miss the window of opportunity for treatment. Despite these advantages mentioned above, the performance of the discrimination model between infertility and pregnancy loss is low. The reason may be due to the overlap of clinical manifestations or the similarity of pathologic mechanisms in some of the two diseases, such as hormonal disorders, thyroid abnormalities and other clinical manifestations in patients with the two diseases. The introduction of other more differentiated indicators may be one of the ways to improve the diagnostic model, which needs further research.

## 5 Limitations

This study has some limitations. Firstly, we investigated the medical records from a single hospital located in one of the more developed cities in western China. Most of the patients and healthy individuals came from city. Due to the longer treatment time, most of the patients who came to our hospital for treatment and physical examination had better living conditions and higher education level. Therefore, the data inevitably have a certain bias. Secondly, vitamin D is affected by factors that include dietary intake and sunlight exposure, among others. Although our study considered daily intake, the levels of vitamin D also fluctuate seasonally, and therefore this potential indicator would need to be validated in different populations and during different seasons, something that was beyond our current means. Lastly, since population lifestyles are largely influenced by the region considered, and more than 99% of the participants in this study were from Western China, caution must be exercised while extrapolating the results to other regions of the world. In our future research, we will expand the number of enrolled participants and cooperate with multiple hospitals in China to verify the effect of our diagnostic model.

## 6 Conclusion

We sought to determine whether combining 25OHVD3 with other clinical indicators could increase its value in the diagnosis of infertility and pregnancy loss. Our results demonstrated that 25OHVD3 was the factor exhibiting the most marked difference between patients with infertility and the control group, and between patients affected by pregnancy loss and the control group. 25OHVD3 has a role in the occurrence and development of infertility. Both of the models we developed using five machine learning algorithms exhibited superior performance. These models are advantageous in that they are relatively simple, as the data can be obtained via routine laboratory analyses. Ultimately, the good performance and high sensitivity of the models presented here may facilitate early detection of infertility and pregnancy loss, in turn enabling timely diagnosis and treatment within the optimal reproductive window. Despite our promising findings, further studies involving larger populations are required to verify the practicality of our models and whether they can yield a clear clinical benefit, as well as the most appropriate methods for stratifying candidate patients.

## Data Availability

The original contributions presented in the study are included in the article/**Supplementary Material**. Further inquiries can be directed to the corresponding author.
