# Supplementary material for: Machine learning algorithm based on combined clinical indicators for the prediction of infertility and pregnancy loss
Source: Front Endocrinol (Lausanne). 2025 Jul 18;16:1544724. doi: 10.3389/fendo.2025.1544724 (PMC12313480; doi:10.3389/fendo.2025.1544724)
Supplement: Supplementary file 1 [file DataSheet1.docx]

**Table S1. Parameters of 979 individuals with different groups**

| **Indicators** | **Mean ± Standard Deviation** | | |  | **P** |  |
| --- | --- | --- | --- | --- | --- | --- |
|  | **1** | **2** | **3** | **1VS2** | **1VS3** | **2VS3** |
| **Age** (year) | 31.15±14.83 | 30.71±14.10 | 30.40±14.72 | 0.876 | 1.000 | 1.000 |
| **HPV52** (human papillomavirus 52) | 0.74±2.45 | 0.51 ±1.12 | 0.55 ±1.31 | 0.028 | 0.052 | 1.000 |
| **HPV83** (human papillomavirus 83) | 0.51±0.13 | 0.52 ±0.14 | 0.53 ±0.13 | 0.013 | 0.123 | 1.000 |
| **PDW** (platelet distribution width) | 13.73±2.40 | 13.85 ±2.78 | 12.93 ±2.73 | 1.000 | 0.000 | 0.000 |
| **BACT** (bacteria in urine, /μL) | 500.88±5170.12 | 324.49 ±641.89 | 1247.85 ±7237.71 | 0.094 | 1.000 | 0.287 |
| **HPV16** (human papillomavirus 16) | 0.54±1.60 | 0.59 ±1.75 | 0.98 ±5.29 | 0.033 | 0.517 | 0.693 |
| **ESR60M** (erythrocyte sedimentation rate) | 11.52±9.98 | 7.00 ±2.90 | 9.45 ±6.29 | 0.000 | 0.000 | 0.002 |
| **EC** (urinary epithelial cell, /μL) | 12.95±25.08 | 22.53 ±37.38 | 29.32 ±49.79 | 0.000 | 0.000 | 0.065 |
| **TPOAb** (thyroid peroxidase antibody, IU/mL) | 24.27±57.11 | 39.63 ±92.19 | 32.40 ±69.48 | 0.000 | 0.001 | 0.507 |
| **SG** (specific gravity) | 1.02±0.01 | 1.02 ±0.01 | 1.36 ±3.10 | 1.000 | 0.038 | 0.045 |
| **HDL** (high density lipoprotein, mmol/L) | 1.44±0.26 | 1.21 ±0.35 | 1.26 ±0.31 | 0.000 | 0.000 | 0.170 |
| **MONO%** (Monocyte, %) | 0.07±0.02 | 0.06 ±0.02 | 0.06 ±0.02 | 0.000 | 0.001 | 1.000 |
| **TBA** (total bile acids, μmol/L) | 2.78±1.85 | 3.08 ±2.93 | 2.94 ±2.61 | 0.245 | 0.849 | 1.000 |
| **ALP** (alkaline phosphatase, IU/L) | 63.12±17.27 | 62.94 ±22.93 | 65.27 ±25.22 | 0.257 | 0.006 | 0.511 |
| **PLT** (platelet count, 10E9/L) | 244.15±52.99 | 244.42 ±70.52 | 244.91 ±64.51 | 0.510 | 0.595 | 1.000 |
| **MPV** (mean platelet volume, fl) | 11.25±1.01 | 11.06 ±1.10 | 10.74 ±1.17 | 0.033 | 0.000 | 0.001 |
| **HPV68** (human papillomavirus 68) | 0.36±0.11 | 0.35 ±0.11 | 0.51 ±1.87 | 1.000 | 0.340 | 0.186 |
| **NEUT%** (neutrophil, %) | 0.58±0.08 | 0.64 ±0.11 | 0.61 ±0.10 | 0.000 | 0.000 | 0.000 |
| **EO%** (eosinophil, %) | 0.02±0.02 | 0.01 ±0.01 | 0.02 ±0.03 | 0.000 | 1.000 | 0.000 |
| **TgZ** (thyroglobulin, ng/mL) | 17.98±44.52 | 12.23 ±20.00 | 11.48 ±13.77 | 0.002 | 0.015 | 1.000 |
| **IBIL** (indirect bilirubin, μmol/L) | 8.37±2.51 | 8.63 ±3.49 | 7.89 ±3.40 | 1.000 | 0.009 | 0.008 |
| **HPV59** (human papillomavirus 59) | 0.30±0.13 | 1.29 ±5.69 | 0.60 ±2.43 | 0.001 | 0.865 | 0.038 |
| **HPV66** (human papillomavirus 66) | 0.58±1.01 | 0.86 ±3.40 | 0.57 ±0.15 | 0.127 | 1.000 | 0.195 |
| **RDW%** (red cell distribution width, %) | 0.13±0.01 | 0.13 ±0.02 | 0.13 ±0.02 | 0.000 | 1.000 | 0.000 |
| **MCH** (mean corpuscular hemoglobin, pg) | 29.70±2.24 | 29.82 ±2.65 | 29.95 ±2.27 | 1.000 | 0.520 | 1.000 |
| **HPV45** (human papillomavirus 45) | 0.41±0.13 | 0.42 ±0.14 | 0.42 ±0.13 | 0.415 | 0.927 | 1.000 |
| **HPV82** (human papillomavirus 82) | 0.37±0.13 | 0.35 ±0.11 | 0.39 ±0.43 | 0.562 | 0.006 | 0.247 |
| **β-globin** (g/L) | 46.32±22.28 | 52.24 ±20.69 | 50.12 ±19.19 | 0.000 | 0.000 | 0.629 |
| **HPV58** (human papillomavirus 58) | 0.41±0.11 | 0.83 ±3.87 | 0.72 ±3.87 | 0.513 | 0.691 | 1.000 |
| **LYMPH%** (Lymphocyte, %) | 0.50±3.02 | 0.28 ±0.10 | 0.31 ±0.09 | 0.357 | 0.740 | 1.000 |
| **TP** (total protein, g/L) | 75.15±4.26 | 75.26 ±5.40 | 76.01 ±4.10 | 0.000 | 0.000 | 0.000 |
| **HPV43** (human papillomavirus 43) | 0.41±0.12 | 0.65 ±3.08 | 0.60 ±3.57 | 0.014 | 1.000 | 0.113 |
| **AST** (aspartate aminotransferase, IU/L) | 17.64±4.72 | 19.41 ±5.95 | 19.51 ±6.37 | 0.677 | 0.987 | 1.000 |
| **PathCAST** (pathologic cast, /μL) | 0.03±0.09 | 0.22 ±0.30 | 0.07 ±0.11 | 0.000 | 0.000 | 1.000 |
| **DBIL** (direct bilirubin, μmol/L) | 5.16±2.52 | 4.69 ±1.95 | 3.97 ±1.94 | 0.040 | 1.000 | 0.053 |
| **T4** (thyroxine, nmol/L) | 97.51±15.70 | 108.16 ±20.61 | 107.14 ±19.67 | 0.000 | 0.005 | 0.000 |
| **E2** (estradiol, pg/mL) | 77.11±83.47 | 154.86±232.87 | 597.03±952.45 | 0.213 | 0.004 | 0.000 |
| **HyCAST** (hyalin cast, /μL) | 0.06±0.15 | 0.06 ±0.08 | 0.23 ±0.24 | 0.000 | 0.000 | 1.000 |
| **RBC** (red blood cell count, 10E12/L) | 4.75±3.02 | 4.37 ±0.40 | 4.46 ±0.44 | 0.788 | 0.000 | 0.000 |
| **HCT** (hematocrit) | 0.41±0.04 | 0.40 ±0.04 | 0.41 ±0.04 | 0.608 | 0.000 | 0.000 |
| **fT4** (free thyroxine, pmol/L) | 16.06±2.17 | 16.85 ±2.35 | 16.60 ±2.39 | 0.001 | 0.002 | 1.000 |
| **HPV81** (human papillomavirus 81) | 0.37±0.13 | 0.38 ±0.21 | 0.38 ±0.12 | 0.000 | 0.008 | 0.001 |
| **HPV18** (human papillomavirus 18) | 0.41±0.14 | 0.55 ±0.64 | 0.57 ±1.57 | 0.000 | 0.022 | 0.468 |
| **MCV** (mean corpuscular volume, fl) | 92.17±5.20 | 90.78 ±6.01 | 91.02 ±5.45 | 0.002 | 0.004 | 1.000 |
| **MONO** (monocyte, 10E9/L) | 0.38±0.12 | 0.42 ±0.17 | 0.39 ±0.13 | 0.251 | 0.132 | 1.000 |
| **HPV61** (human papillomavirus 61) | 0.47±0.11 | 0.91 ±4.41 | 1.41 ±5.38 | 0.000 | 0.000 | 1.000 |
| **TBIL** (total bilirubin, μmol/L) | 12.44±3.74 | 13.17 ±4.62 | 12.15 ±4.96 | 0.009 | 1.000 | 0.021 |
| **T3** (triiodothyronine, nmol/L) | 1.78±0.27 | 1.96 ±0.37 | 1.95 ±0.37 | 0.046 | 1.000 | 0.289 |
| **RDW** (red cell distribution width, fl) | 43.33±3.43 | 43.94 ±4.17 | 42.84 ±4.05 | 0.512 | 0.009 | 0.349 |
| **EO** (eosinophil, 10E9/L) | 0.10±0.08 | 0.08 ±0.08 | 0.10 ±0.13 | 1.000 | 0.035 | 0.012 |
| **aTg** (anti-thyroglobulin antibody, IU/mL) | 40.53±93.87 | 70.21 ±192.60 | 72.81 ±278.84 | 0.000 | 0.000 | 1.000 |
| **LDL** (low-density lipoprotein, mmol/L) | 2.50±0.52 | 2.53±0.70 | 2.14±0.68 | 0.503 | 0.072 | 0.001 |
| **HPV31** (human papillomavirus 31) | 0.45±0.29 | 0.70 ±2.84 | 0.41 ±0.14 | 0.010 | 1.000 | 0.018 |
| **UA** (uric acid, μmol/L) | 278.44±52.61 | 293.91 ±76.97 | 293.20 ±73.58 | 0.090 | 0.055 | 1.000 |
| **TG** (triglycerides, mmol/L) | 1.18±1.20 | 1.74 ±1.62 | 1.25 ±0.75 | 0.006 | 0.000 | 0.000 |
| **NonSEC** (non squamous epithelial cell, /μL) | 1.06±1.70 | 1.16 ±1.13 | 3.23 ±3.71 | 1.000 | 0.076 | 0.461 |
| **HPV55** (human papillomavirus 55) | 0.44±0.27 | 0.46 ±0.54 | 0.52 ±2.03 | 0.007 | 0.010 | 1.000 |
| **TESTO** (testosterone, ng/dL) | 0.36±0.78 | 0.30 ±0.19 | 0.36 ±0.64 | 0.000 | 0.000 | 0.000 |
| **G** (globin, g/L) | 29.38±2.85 | 30.06 ±3.75 | 30.34 ±3.35 | 1.000 | 0.000 | 0.000 |
| **25OHVD3** (25-hydroxy vitamin D3, ng/mL) | 33.28±6.19 | 14.10 ±7.75 | 15.80 ±8.82 | 1.000 | 1.000 | 1.000 |
| **HPV11** (human papillomavirus 11) | 0.39±0.15 | 0.35 ±0.14 | 0.37 ±0.15 | 0.129 | 0.000 | 0.188 |
| **HPV44** (human papillomavirus 44) | 0.44±0.13 | 0.65 ±2.33 | 0.56 ±3.23 | 0.774 | 0.085 | 0.886 |
| **UPH** (acidity) | 6.04±0.61 | 6.36 ±0.84 | 6.05 ±0.79 | 0.000 | 0.000 | 0.018 |
| **MUCUS** (mucus, /μL) | 7.52±28.57 | 0.51 ±0.57 | 1.42 ±2.95 | 0.197 | 1.000 | 0.245 |
| **HPV56** (human papillomavirus 56) | 0.49±1.35 | 0.91 ±3.77 | 0.71 ±3.65 | 0.463 | 1.000 | 1.000 |
| **CysC** (Cystatin C, mg/L) | 0.75±0.11 | 0.73 ±0.13 | 0.69 ±0.12 | 0.000 | 1.000 | 0.000 |
| **A/G** (albumin/globulin) | 1.59±0.18 | 1.53 ±0.26 | 1.55 ±0.21 | 0.000 | 0.000 | 1.000 |
| **CHO** (cholesterol, mmol/L) | 4.31±0.63 | 4.38 ±0.80 | 4.23 ±0.79 | 1.000 | 1.000 | 1.000 |
| **ALB** (albumin, g/L) | 45.87±2.88 | 45.21 ±4.02 | 45.91 ±3.88 | 0.459 | 0.000 | 0.000 |
| **HPV53** (human papillomavirus 53) | 0.45±0.15 | 0.57 ±1.26 | 0.61 ±2.52 | 0.001 | 0.048 | 0.764 |
| **BASO** (basophils, 10E9/L) | 0.03±0.01 | 0.03 ±0.01 | 0.03 ±0.03 | 0.914 | 1.000 | 0.183 |
| **GGT** (gamma-glutamyl transferase, IU/L) | 15.90±7.15 | 19.01 ±13.01 | 18.62 ±11.41 | 0.021 | 1.000 | 0.023 |
| **25OHVD2 (25-hydroxy vitamin D2)** | 0.96±1.00 | 1.55 ±1.93 | 1.34 ±1.51 | 0.000 | 0.030 | 0.032 |
| **HPV35 (human papillomavirus 35)** | 0.38±0.11 | 0.39 ±0.10 | 0.39 ±0.12 | 1.000 | 0.644 | 1.000 |
| **BU (Urea, mmol/L)** | 4.54±1.02 | 4.15 ±0.97 | 4.72 ±1.22 | 0.796 | 1.000 | 0.384 |
| **PT (prothrombin time, s)** | 10.98±0.86 | 10.47 ±0.55 | 10.56 ±0.56 | 0.000 | 0.000 | 1.000 |
| **25OHVD (25-hydroxy vitamin D,** ng/mL**)** | 34.27±6.25 | 15.97 ±8.33 | 17.14 ±8.92 | 1.000 | 0.192 | 0.554 |
| **NEUT** (neutrophil, 10E9/L) | 3.54±0.79 | 4.64 ±2.38 | 3.91±1.64 | 0.291 | 0.096 | 1.000 |
| **FT3** (free triiodothyronine, pmol/L) | 4.73±0.55 | 5.15 ±0.69 | 5.04 ±0.66 | 1.000 | 0.000 | 0.000 |
| **HPV33** (human papillomavirus 33) | 0.42±0.69 | 0.48 ±0.78 | 0.38 ±0.11 | 0.000 | 0.000 | 0.116 |
| **WBC** (white blood cell count, 10E9/L) | 6.70±4.09 | 6.56±2.14 | 6.09 ±1.69 | 0.000 | 0.000 | 0.024 |
| **HCY** (homocysteine, μmol/L) | 12.16±6.95 | 11.31 ±5.12 | 9.38 ±4.55 | 0.000 | 0.000 | 0.202 |
| **PLCR** (platelet large cell ratio, %) | 0.35±0.08 | 0.33 ±0.10 | 0.31 ±0.10 | 0.000 | 0.000 | 0.000 |
| **AST/ALT** | 1.22±0.37 | 1.19 ±0.37 | 1.24 ±0.46 | 0.000 | 0.000 | 0.073 |
| **GLU** (glucose, mmol/L) | 4.87±0.36 | 5.19 ±1.79 | 5.09 ±0.58 | 0.000 | 0.000 | 1.000 |
| **HPV39** (human papillomavirus 39) | 0.39±0.23 | 0.43 ±1.15 | 0.39 ±0.13 | 0.102 | 0.002 | 0.628 |
| **HPV06** (human papillomavirus 06) | 0.41±0.12 | 0.62 ±2.53 | 0.73 ±4.58 | 1.000 | 0.084 | 0.081 |
| **HPV42** (human papillomavirus 42) | 0.42±0.13 | 0.39 ±0.11 | 0.44 ±0.54 | 0.000 | 0.015 | 0.001 |
| **LYMPH** (Lymphocyte, 10E9/L) | 1.87±0.48 | 1.79 ±0.66 | 1.84 ±0.54 | 0.003 | 0.000 | 0.000 |
| **TSH** (thyroid stimulating hormone, μIU/mL) | 2.46±1.03 | 2.43 ±1.33 | 2.49 ±1.27 | 0.107 | 0.425 | 1.000 |
| **FSH** (follicle-stimulating hormone, mIU/mL) | 10.28±17.62 | 6.75 ±2.26 | 9.57 ±6.07 | 0.002 | 0.000 | 0.292 |
| **HPV26** (human papillomavirus 26) | 0.39±0.10 | 0.37 ±0.13 | 0.39 ±0.12 | 1.000 | 0.340 | 0.419 |
| **BASO%** (basophils, %) | 0.01±0.00 | 0.00 ±0.00 | 0.01 ±0.00 | 0.001 | 0.032 | 0.671 |
| **ALT** (alanine aminotransferase, IU/L) | 16.04±8.56 | 24.17 ±13.47 | 18.68 ±10.50 | 1.000 | 1.000 | 1.000 |
| **CRE** (creatinine, μmol/L) | 57.55±9.93 | 52.92 ±13.63 | 51.94 ±9.84 | 1.000 | 0.513 | 1.000 |
| **TT** (Thrombin Time, s) | 18.01±1.23 | 17.15 ±1.19 | 17.63 ±1.37 | 0.203 | 0.020 | 0.000 |
| **HGB** (Hemoglobin, g/L) | 132.45±12.73 | 131.34 ±13.59 | 132.83 ±15.14 | 0.366 | 1.000 | 0.199 |
| **LH** (luteinizing hormone, mIU/mL) | 14.41±15.86 | 9.19 ±12.50 | 7.01 ±10.45 | 0.003 | 0.106 | 0.656 |
| **DDi** (D-Dimer, mg/L) | 0.39±0.51 | 0.37 ±0.45 | 0.39 ±0.50 | 0.698 | 1.000 | 1.000 |
| **HPV51** (human papillomavirus 51) | 0.37±0.11 | 0.48 ±1.09 | 0.47 ±0.72 | 0.001 | 0.024 | 1.000 |
| **HPV40** (human papillomavirus 40) | 0.32±0.12 | 0.32 ±0.11 | 0.34 ±0.12 | 0.000 | 0.001 | 0.032 |
| **MCHC** (Mean Corpuscular Hemoglobin Concentration, g/L) | 322.18±11.67 | 325.74 ±16.93 | 328.11 ±13.21 | 0.028 | 1.000 | 0.122 |
| **PROG** (Progesterone, ng/mL) | 3.78±7.29 | 8.72 ±11.69 | 9.73 ±12.58 | 0.000 | 0.137 | 0.000 |

1: female controls (n=327), 2: female patients with failed pregnancy (n=319), 3: female patients with infertility (n=333)

P value < 0.025 was considered significant.

**Table S2. Logistic Regression Analysis of Predictive Factors for infertility**

| **Indicators** | **P** | **OR** | **95% C.I.for OR** | |
| --- | --- | --- | --- | --- |
|  |  |  | **Lower** | **Upper** |
| ESR60M | 0.000 | 0.884 | 0.831 | 0.941 |
| HD | 0.002 | 0.091 | 0.019 | 0.428 |
| PT | 0.000 | 0.106 | 0.044 | 0.252 |
| TT | 0.041 | 0.802 | 0.649 | 0.991 |
| LH | 0.019 | 0.964 | 0.935 | 0.994 |
| 25OHD3 | 0.000 | 0.833 | 0.792 | 0.877 |
| CysC | 0.029 | 0.081 | 0.009 | 0.773 |
| BU | 0.302 | 1.173 | 0.867 | 1.588 |
| ACA | 0.000 | 1.701 | 1.228 | 2.049 |
| HCY | 0.005 | 0.942 | 0.903 | 0.982 |
| CRE | 0.005 | 0.970 | 0.950 | 0.991 |
| MCHC | 0.001 | 1.027 | 1.010 | 1.043 |
| GGT | 0.001 | 1.115 | 1.044 | 1.192 |
| MUCUS | 0.001 | 0.902 | 0.848 | 0.959 |
| EC | 0.128 | 1.030 | 0.991 | 1.071 |
| Globin | 0.000 | 0.972 | 0.957 | 0.987 |
| TG | 0.000 | 1.332 | 1.032 | 2.089 |
| E2 | 0.002 | 1.003 | 1.001 | 1.005 |
| CEA | 0.888 | 1.038 | 0.615 | 1.753 |
| AST | 0.003 | 1.148 | 1.049 | 1.256 |


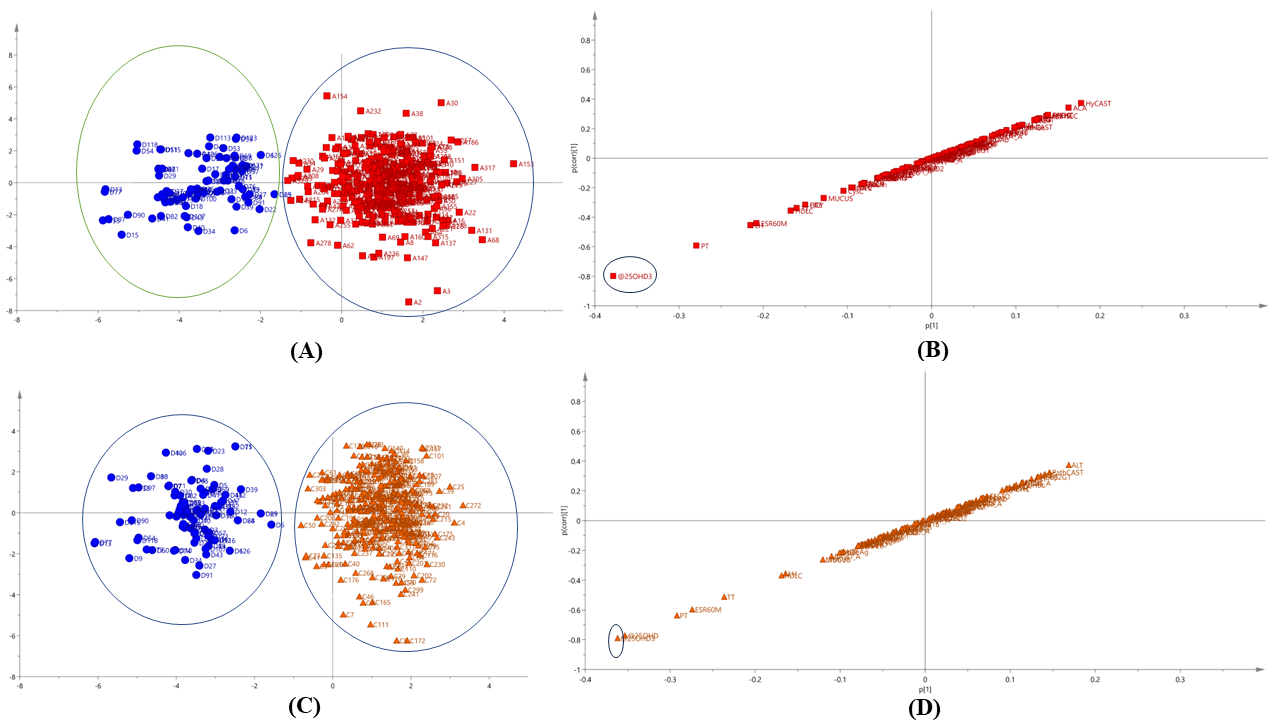


Figure S1. Multivariate data analysis. (A) Score plots of the supervised OPLS-DA model for the control group (blue) and female infertility group (red). (B). S-plot analysis of the OPLS-DA model derived from the control group (blue) and female infertility group (red). (C) Score plots of the supervised OPLS-DA model for the control group (blue) and female pregnancy loss group (orange). (D). S-plot analysis of the OPLS-DA model derived from the control group (blue) and female infertility group (orange).
